# In Vitro Assessment of Gut Microbiota Modulation Through Functional Biscuits Enriched with Almond By-Products

**DOI:** 10.3390/foods15020313

**Published:** 2026-01-15

**Authors:** Angela Racioppo, Maria Rosaria Corbo, Angela Guerrieri, Milena Sinigaglia, Antonio Bevilacqua, Rossella Caporizzi, Antonio Derossi, Barbara Speranza

**Affiliations:** Department of Agriculture, Food, Natural Resources and Engineering, University of Foggia, Via Napoli 25, 71122 Foggia, Italy; angela.racioppo@unifg.it (A.R.); angela.guerrieri@unifg.it (A.G.); milena.sinigaglia@unifg.it (M.S.); antonio.bevilacqua@unifg.it (A.B.); rossella.caporizzi@unifg.it (R.C.); antonio.derossi@unifg.it (A.D.); barbara.speranza@unifg.it (B.S.)

**Keywords:** almond skin, prebiotics, gut microbiota, functional foods, food by-products

## Abstract

Almond skin is an abundant by-product of almond processing and is recognized for its rich content of dietary fiber, polyphenols, and unsaturated fatty acids along with potential health benefits. This study aimed to evaluate the nutritional composition, prebiotic potential, and microbiota modulation properties of dehydrated almond skin, including its use in 3D-printed functional biscuits. Nutritional analysis revealed high dietary fiber (62.6%) and substantial antioxidant capacity linked to polyphenols. Almond skin supplementation with a concentration ranging from 2.5% to 5.0% significantly enhanced the viability of various probiotic strains during storage, extending their shelf life. Two biscuit formulations, with and without almond skin, were produced and subjected to simulated gastrointestinal digestion (INFOGEST protocol) followed by in vitro fermentation using a minimal gut microbiota model (*Bifidobacterium longum*, *Lactobacillus rhamnosus*, *Bacteroides caccae*, *Escherichia coli*, *Segatella copri*, and *Clostridioides difficile*). Results demonstrated that biscuit enriched with almond skin selectively promoted the growth of beneficial bacteria such as *B. longum* and *L. rhamnosus* (from 6.9 to 8.5 log cfu/mL and from 7.8 to 9.0 log cfu/mL, respectively) while suppressing pathogens including *C. difficile* and *E. coli*. Moreover, enriched biscuits retained higher polyphenol content and exhibited a favorable macronutrient profile. These findings support the valorization of almond skin as a sustainable functional ingredient offering prebiotic effects and probiotic viability protection, with promising applications in personalized nutrition and gut health management. Further in vivo studies and clinical trials are necessary to confirm these effects and optimize formulations for commercial use.

## 1. Introduction

The past decade has witnessed a paradigm shift in food science and nutrition, emphasizing sustainable approaches to food production alongside advances in human health promotion. Functional foods, designed to deliver health benefits beyond basic nutrition, have gained considerable attention for their potential to reduce disease risk and improve physiological functions [1]. A critical focus regarding impacts is the valorization of food processing by-products, which offers an innovative route to reduce food waste, mitigate environmental impacts, and develop nutritionally enhanced products in accordance with circular economy principles [2].

Almonds (*Prunus dulcis*) occupy a prominent place in this narrative due to their widespread consumption, palatability, and dense nutrient profile, which includes lipids rich in monounsaturated fats, plant proteins, vitamins, minerals, and a complex array of phytochemicals [3]. With global almond production exceeding 1.3 million metric tons annually, significant quantities of by-products such as almond skins are generated during industrial processing, which until recently were discarded or underutilized [4]. These skins, constituting up to 4–7% of the kernel weight, represent a rich reservoir of dietary fibers (approximately 45% by weight), polymerized polyphenols (notably proanthocyanidins), and fermentable carbohydrates, including xylo-oligosaccharides and hemicelluloses, all of which confer notable prebiotic and bioactive properties [3].

The gut microbiota has emerged as a central player in human health, contributing to nutrient metabolism, immune regulation, and the pathogenesis of metabolic and inflammatory diseases. Prebiotics, defined as substrates selectively utilized by host microorganisms conferring health benefits, have been identified among dietary fibers and polyphenols that escape digestion in the upper gastrointestinal tract and reach the colon where they undergo fermentation by the gut microbiota [5].

Almond skin components fit this prebiotic profile and have been reported to stimulate beneficial bacterial genera such as *Bifidobacterium*, *Lactobacillus*, while suppressing pathogenic species including *Clostridium perfringens* and *Escherichia coli* in various in vitro and in vivo studies [3].

Mechanistically, fermentation of almond skin polyphenols and fibers leads to increased short-chain fatty acid (SCFA) production, especially butyrate, which supports colonocyte health, enhances the mucosal barrier, and exerts anti-inflammatory effects [6,7]. Clinical trials have reinforced these findings by documenting increased microbial diversity and enrichment of beneficial bacteria, including *Bifidobacterium*, *Roseburia*, and *Faecalibacterium prausnitzii*, following almond consumption, indicating its role as a prebiotic substrate capable of modulating the composition and activity of the gut microbiota [7]. However, most previous studies have focused on whole almond or almond flour, with limited investigation into specific prebiotic potential of almond skins as isolated functional ingredients, particularly when incorporated into novel food matrices.

In parallel with advances in microbiota research, innovative food technologies such as 3D food printing have opened promising avenues such as future development of personalized functional foods. This approach has the potential to facilitate the incorporation of bioactive ingredients and food by-products into structured, customized delivery systems that could promote sustainability and reduce waste while enabling precise tailoring of nutritional profiles to meet individual health needs [8]. The integration of almond skins within 3D-printed food products offers a novel platform to harness their prebiotic potential while advancing sustainable food design aligned with contemporary nutritional paradigms and circular economy principles.

Despite the recognized nutritional value of almond by-products, their application as functional ingredients in advanced food manufacturing systems remains underexplored. This study addresses this gap through a novel integrated approach that combines: (1) comprehensive nutritional and bioactive characterization of almond skin as an isolated functional ingredient; (2) incorporation into 3D-printed biscuits as an innovative delivery system for sustainable food design, and (3) validation using a defined minimal gut microbiota model (HGMM) under simulated gastrointestinal conditions. This combined methodology enables precise mechanistic investigation of prebiotic effects while advancing both circular economy principles and personalized nutrition. Through this integrated approach, this research aims to provide a proof-of-concept for the sustainable valorization of almond by-products as functional ingredients capable of beneficially modulating gut microbiota, demonstrating the feasibility of this approach and supporting the development of personalized functional foods.

## 2. Materials and Methods

### 2.1. Raw Materials

The almond cultivar Filippo Cea grown in the “agro Manfredonia” area, situated between Foggia and Manfredonia (Italy), was used in this study and sourced from a local company located in Foggia (Arcadia s.a.s., Foggia, Italy). The almonds were sorted and blanched in hot water for 1 min. The wet skins were collected and immediately transported into polyethylene bags for food matrices to the laboratory for dehydration at 40 °C for 120 min using a laboratory-scale air drier (Klarstein, Arizona Jerky 500W, Chal-Tec GmbH, Mühlenstraße, Germany) until water activity below 0.4 was reached to ensure microbiological stability. The dried skins were then ground by using a mixer (Grindomix GM 200, Retsch, Haan, Germany) for 8 s at 10,000 rpm to obtain a powder with particle size <600 µm and then stored in glass bottles with screw caps at room temperature until use for further analysis.

Icing sugar (Eridania, Bologna, Italy), and almond flour (Arcadia Mandorle, Foggia, Italy) were purchased from local retailers.

### 2.2. Nutritional and Bioactive Characterization of Raw Materials

The nutritional properties of dehydrated almond skin were evaluated by proximate analysis according to AOAC-standard methods, including protein (AOAC 984.13), fat (AOAC 983.23), fiber (AOAC 991.43), and carbohydrate content (by difference). Mineral content was determined according to EN 13805:2014 [9]. To identify the main source of bioactive compounds and compare the phenolic potential of almond skin with almond flour, TPC and antioxidant activity were determined on both raw materials following the extraction and analytical procedures described in Section 2.3. This comparison was essential to establish whether the prebiotic effects observed could be specifically attributed to almond skin components.

### 2.3. Total Phenol Content and Antioxidant Activity

Phenolic compounds were extracted from raw materials (almond flour and almond skin powder) following la Gatta et al.’s procedure [10]. One gram of each sample was extracted with 5 mL of methanol/HCl 99:1 (*v*/*v*) for 20 min at room temperature in a sonicator bath (Sonica Ultrasonic cleaner; Soltec, Milano, Italy). Samples were left at room temperature for approximately 15 min and centrifuged at 7500× *g* for 10 min at 7 °C. Extraction was performed in triplicate, and supernatants were collected in a 20 mL flask and stored at −20 °C until analysis.

Total phenol content (TPC) was quantified using the Folin–Ciocalteu method [11]. A mixture of 980 μL of deionized water, 20 μL of sample extract, and 100 μL Folin–Ciocalteu reagent was prepared, followed by the addition of 800 μL 7.5% Na_2_CO_3_ after 3 min. Samples were incubated in the dark at room temperature for 60 min. Absorbance was measured at 720 nm using a UV–Vis spectrophotometer (Thermo Fisher Scientific, Waltham, MA, USA). Results were expressed as mg of gallic acid equivalents (GAE) per gram of sample. Measurements were performed in triplicate.

Antioxidant activity was assessed via the DPPH assay, where 50 μL of extract was added to 950 μL DPPH solution. Absorbance was recorded at 517 nm after 30 min in the dark. Results were expressed as μmol Trolox equivalents (TE) per gram of sample. Measurements were performed in triplicate.

### 2.4. Effect of Almond Skin on Strains’ Cultivability

#### 2.4.1. Microorganisms

Five microorganisms were tested: *Lactobacillus acidophilus* (DSM 20079), *Lactobacillus delbruekii* subsp. *bulgaricus* (DSM 20081), *Streptococcus thermophilus* (DSM 20479), *Bifidobacterium longum* (DSM 20219), and *Lactiplantibacillus plantarum* (DSM 1055). Cultures were stored at −20 °C in MRS broth (Oxoid, Milan, Italy) with 33% of sterile glycerol (J.T. Baker, Milan, Italy) or in MRS broth supplemented with 0.5% cysteine (cMRS) (Sigma-Aldrich, Milan, Italy). Strains were grown anaerobically in their respective media at 37 °C for 24 h before assays. Cells were centrifuged at 4000× *g* for 10 min, washed, and resuspended in sterile saline solution, with an initial concentration around 9 log cfu/mL.

#### 2.4.2. *In Vitro* Evaluation of Prebiotic Compounds

Sterile tubes containing 75 mL of Reconstructed Skim Milk powder (10%, RSM, Oxoid) were supplemented with dehydrated almond skins at 2.5%, 5.0% and 7.5% (*w*/*v*). Each tube was inoculated with a single microorganism at about 8.0 log cfu/mL. RSM without almond skin was used as a control. Samples were stored at 4 °C, 15 °C, and 30 °C for 90 days. Viability was assessed at 10-day intervals by pour-plating on MRS agar or cMRS agar (for *Bifidobacterium*), with anaerobic incubation at 37 °C for 48 h. Experiments were performed in duplicate across two batches. Data were modelled using modified Weibull equations [12]:

(1) $$N\;=\;N_{0}\;-\;{\left(t/\sigma \right)}^{p}$$

where *N* and *N*_0_ (log cfu/mL) are the cell count throughout the time and the initial cell count, respectively; σ is the first reduction time (days), i.e., the time to attain a reduction of 1 log cfu/mL; and *p* is the shape parameter (*p* < 1 upward curve; *p* > 1 downward curve).

Data were also fitted through the Weibull equation modified by Bevilacqua et al. [13] to evaluate death time:

(2) $$\left(N/No\right)=1-{\left(t/d.t.\right)}^{p}$$

where *d.t.* is the death time (days) of the microbial population.

Using the fitting parameters, Sl-7 and Sl-6 (time in days to attain a cell count of 7 and 6 cfu/mL, respectively) were calculated:

(3) $$Sl=\sigma \times {\left(N_{0}-L_{c}\right)}^{1}/p$$

where *L_c_* is the critical limit (7 or 6 log cfu/mL), considered as a “probiotic shelf-life limit”. The fitting was carried out using Statistica for Windows, version 12.0 (Statsoft, Tulsa, OK, USA).

The quality of Weibull model fits was evaluated using coefficient of determination (R^2^) and root mean square error (RMSE). All model achieved R^2^ > 0.95 and RMSE < 0.3 log cfu/mL, indicating excellent fit to the experimental data. When viable counts remained above the detection limit (1 log cfu/mL) at the end of the observation period, death times were extrapolated and reported as >500 days to acknowledge the uncertainty associated with long-term predictions beyond the experimental timeframe.

### 2.5. Preparation and Characterization of Three-Dimensional-Printed Biscuits

#### 2.5.1. Preparation of Three-Dimensional-Printed Biscuits

Two biscuit formulations were developed based on a recipe adapted from Ukkunda et al. [14] to ensure a good printability of the dough: a control formulation (F0) consisting of almond flour (68.19 g/100 g), icing sugar (15.50 g/100 g), water (16.31 g/100 g); and an enriched formulation (F1) containing almond flour (64.69 g/100 g), icing sugar (15.50 g/100 g), almond skin powder (3.50 g/100 g), and water (16.31 g/100 g). The concentration of 3.5% almond skin was selected as an intermediate value based on the viability studies, which indicated optimal probiotic protection at concentration between 2.5–5.0% without inhibitory effects at higher concentrations. All ingredients were mixed using a planetary kneader (Bella Mini Kitchen machine, Klarstein, Berlin, Germany) for 5 min at speed 2, wrapped in cling film, and rested at 25 °C for 20 min before printing experiments. For 3D printing step, a cylindric 3D virtual model has been created using computer-aided design (CAD) software (TinkerCad, Autodesk Inc., San Rafael, CA, USA) with 35 mm of diameter and height of 6.6 mm. The CAD models were exported as .stl files and processed with a slicing software, CURA v5.0.0, to set the printing parameters. Printing parameters were optimized through preliminary printability tests aimed at ensuring extrusion continuity, shape fidelity and structural stability of the dough during deposition. These tests involved systematic adjustments of various printing variables, taking into account the rheological properties of the formulations. Printability and printing fidelity were qualitatively evaluated by visual inspection and image-based analysis of the printed samples, focusing on the absence of collapse or spreading and on the preservation of the designed geometry. Based on these criteria, a nozzle diameter of 1 mm and an infill density of 40% were selected.

Three-dimensional printing was carried out using a Felix Food Switch printer (IJsselstein, The Netherlands) with the following additional settings: a print speed of 800 mm/min, a layer height of 0.8 mm, a temperature of 30 °C, an extrusion multiplier of 1.2%, a retraction speed of 300 mm/min, and a travel speed of 800 mm/min. Baking was carried out in a static oven (mod. MKF 4642 TS) (EKA, Padova, Italy) at 150 °C for 18 min.

#### 2.5.2. Nutritional and Physical Characteristics of 3D-Printed Biscuits

The water activity of the 3D-printed biscuits was determined using a dew point system (AquaLab 4 TE, Labcell Ltd., Hampshire, UK) previously calibrated with standard solutions. Moisture content (%) was measured gravimetrically according to the AOAC 925.10 method. Analyses were performed in triplicate on each sample.

The nutritional properties of biscuits were evaluated by proximate analysis according to AOAC standard methods, including protein (AOAC 984.13), fat (AOAC 983.23), fiber (AOAC 991.43), and carbohydrate content (by difference).

To evaluate the retention of bioactive compounds throughout the manufacturing process and their potential availability for gut microbiota fermentation, TPC and antioxidant activity were determined on both raw dough and baked biscuit samples (F0 and F1) following the extraction and analytical procedures described in Section 2.3. This assessment pursued to verify whether thermal processing affected the bioactive potential and to confirm that phenolic compounds in the final product could contribute to the prebiotic effects observed in the colonic fermentation model.

### 2.6. Effect of Almond Skin on the Human Gut Microbiota Model

#### 2.6.1. Bacterial Strains, Cultivation Medium, Growth Conditions

Six human gut microbiota representative strains [15,16,17] were obtained from DSMZ (Deutsche Sammlung von Mikroorganismen und Zellkulturen) to construct a Human Gut Microbiota Model (HGMM) (Table 1). Pure strains and the HGMM consortium were cultured anaerobically in Modified Gifu Anaerobic Medium Broth (mGAM Broth, HyServe, Uffenheim, Germany) supplemented with 0.1% *w*/*v* resazurin as an anaerobiosis indicator. Cultures were incubated under oxygen-free conditions using Oxyrase for broth (Sigma-Aldrich, Zwijndrecht, The Netherlands) at 37 °C for 48 h in Hungate tubes, as previously described [18], using mGAM broth. The final pH was adjusted to 7.3 of the media, after sterilization by autoclaving at 115 °C for 15 min, reflecting intestinal host sites relevant for microbial metabolism [19,20]. Strains were stored at −20 °C in glycerol/mGAM (50% *v*/*v*).

#### 2.6.2. Construction of the Human Gut Microbiota Model (HGMM)

Each strain was cultured separately (5 mL mGAM) under anaerobic conditions and incubated to the exponential growth phase (5% *v*/*v*). After 48 h at 37 °C, equal volumes (1 mL) of each strain were pooled to form the consortium in a 100 mL volume. The consortium underwent three subcultures (C1, C2, and C3) to establish stability.

#### 2.6.3. *In Vitro* Gastrointestinal Digestion (INFOGEST Protocol)

Biscuit samples (F0 and F1) and sterile water as a negative control underwent simulated digestion following the INFOGEST protocol [21] with minor modifications, as summarized in Table 2.

Human salivary α-amylase, porcine pepsin, porcine pancreatin, and bovine bile were purchased from Sigma-Aldrich (The Netherlands). The simulation covered three phases, each characterized by distinct conditions and enzymatic processes, beginning with a mastication step conducted using a Seward Stomacher Lab Blender 400 (Seward, Worthing, UK) for 1 min, which mechanically reduced the biscuit samples to mimic chewing before saliva exposure. The oral phase (2 min, pH 7) involved exposure of the samples to simulated salivary fluid (SSF) containing α-amylase; the gastric phase (2 h, pH 3) consisted of combining the oral bolus with simulated gastric fluid (SGF) containing pepsin; and the intestinal phase (2 h, pH 7) involved mixing the gastric chyme with simulated intestinal fluid (SIF) containing pancreatin and bile salts.

After digestion, samples were centrifuged (4000× *g*, 10 min, 4 °C) to separate the undigested pellet and soluble fractions, which were stored at −20 °C for subsequent fermentation. The undigested solid residue represents the non-digestible fraction available for microbial fermentation in the colon.

#### 2.6.4. *In Vitro* Colonic Fermentation

Colonic fermentation was performed anaerobically at 37 °C for 48 h using the undigested solid residues obtained from 5 g of biscuit following simulated gastrointestinal digestion in 500 mL of mGAM medium containing 0.1% resazurin, supplemented with 10% (*v*/*v*) of the soluble intestinal fraction to best mimic colonic conditions [22]. The HGMM consortium was inoculated with equal volumes of each strain cultured to exponential phase, resulting in initial densities of approximately 6–8 log CFU/mL depending on the species. The pH was monitored at each sampling timepoint (0, 6, 24, 48 h) but not actively controlled during fermentation, reflecting the natural acidification resulting from microbial metabolism. Initial pH was 7.3 ± 0.1, and final pH values ranged from 5.8 ± 0.1 to 6.2 ± 0.1 depending on the fermentation substrate.

Fermentation was conducted under an anaerobic chamber. Samples were collected at 0, 6, 24, and 48 h for microbial enumeration on selective media specific for each HGMM strain (Table 1).

#### 2.6.5. Statistical Analysis

The experiments were performed on two independent batches. Data are expressed as mean ± standard deviation. Statistical comparisons were performed as follows: for viability studies (Section 3.2), one-way ANOVA followed by Tukey’s post hoc test was used to compare the effects of different almond skin concentrations (CNT, 2.5%, 5.0%, 7.5%) on each probiotic strain. For fermentation studies (Section 3.3.2), comparison between treatments (CNT, F0, F1) were performed separately for each bacterial strain at each timepoint (6, 24, 48 h) using one-way ANOVA with Tukey’s post hoc test. Statistical significance was set at *p* < 0.05. No correction for multiple testing was applied given the exploratory nature of this minimal consortium study Statistic was made through the software Statistica for Windows version 12.0 (Stasoft, Tulsa, OK, USA).

## 3. Results

### 3.1. Nutritional Composition of Dehydrated Almond Skin

The nutritional analysis (Table 3) revealed that dehydrated almond skin used in this study is an excellent source of dietary fiber and fat. The material exhibited remarkably high dietary fiber content (62.60 ± 12.10%), significant fat content (16.10 ± 0.60%), and moderate protein levels (11.75 ± 1.40%). Beyond macronutrients, almond skin demonstrated substantial concentrations of essential micronutrients and bioactive compounds. Among minerals, notably high levels of zinc (83.70 ± 13.80 mg/kg) and iron (62 ± 11 mg/kg) were detected. From a functional perspective, the almond skin exhibited an exceptional TPC of 38.32 ± 0.04 mg GAE/g, translating into substantial antioxidant capacity as confirmed by DPPH scavenging activity of 5.08 ± 0.02 µmol TE/g. In comparison, almond flour showed significantly lower bioactive compound concentrations, with TPC of 2.57 ± 0.03 mg GAE/g and DPPH activity of 1.7 ± 0.02 µmol TE/g.

The fatty acid composition (Table 4) revealed that the lipid fraction of the dehydrated almond skin consists primarily of unsaturated fatty acids (UFA), which constitute approximately 86.90% of total fat. This fraction comprises both monounsaturated fatty acids (MUFA) and polyunsaturated fatty acids (PUFA). Specifically, MUFAs dominate the profile, accounting for 47.92 ± 6.36% of total fat, with oleic acid representing the predominant fatty acid at 47.14 ± 6.36%. PUFAs account for 38.98 ± 5.18%, mainly constituted by linoleic acid (omega-6) at 38.34 ± 5.18%, while alpha-linolenic acid (omega-3) content is lower at 0.64 ± 0.11%. Saturated fatty acids (SFA) form the minority component, totaling 13.10 ± 1.46%, with palmitic acid as the most abundant saturated fatty acid (10.05 ± 1.42%)

### 3.2. Effect of Almond Skin on Strains Cultivability

The protective effect of almond skin on microbial viability was evaluated through death kinetics studies at different storage temperatures. At low temperatures (4 °C and 15 °C), almond skin supplementation maintained final cell concentrations of 8.6 and 8.7 log cfu/mL, respectively, with no significant differences observed between supplemented samples. In contrast, the control group at 4 °C declined to 7 log cfu/mL after 90 days. Overall, almond skin supplementation exerted a protective effect on microbial viability across all tested conditions.

**Figure 1:**
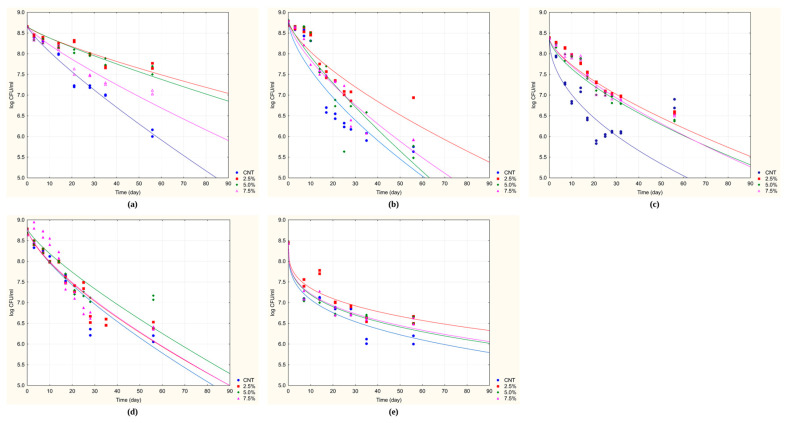
Death kinetics of probiotic strains inoculated in Reconstructed Skim Milk supplemented with almond skin and stored at 30 °C for 90 days. (**a**) *B. longum* DSM 20219; (**b**) *L. acidophilus* DSM 20079; (**c**) *L. plantarum* DSM 1055; (**d**) *L. delbruekii* subsp. *bulgaricus* DSM 20081; (**e**) *S. thermophilus* DSM 20479. Data points are the mean of two independent repetitions ± standard deviation. Solid lines represent the best fit of the data using the Weibull equation.

At 30 °C, initial cell counts were consistently high, ranging from approximately 8.4 to 8.8 log cfu/mL across all strains. For the probiotic strain *B. longum* DSM 20219, the control group showed the sharpest decline in viability, falling below the critical threshold of 6.0 log cfu/mL around day 55. In contrast, all samples supplemented with almond skin (2.5%, 5.0%, and 7.5%) maintained viability above this threshold throughout most of the storage period. Notably, the sample supplemented with 5.0% almond skin retained counts at or above 6.0 log cfu/mL even on day 70 (Figure 1a).

A similar protective trend was observed for *L. acidophilus* DSM 20079 (Figure 1b). The control viability dropped below 6.0 log cfu/mL by day 40, whereas all supplemented samples sustained viable counts above this threshold until after day 60, indicating a statistically significant enhancement of survival due to almond skin addition.

Among *L. plantarum* DSM 1055, the control group showed significant instability, with counts dropping below 6.0 log cfu/mL around day 35. The greatest protective effect was achieved by supplementing with 2.5% almond skin, which demonstrated the slowest decline in viability and maintained counts above 6.30 log cfu/mL until approximately day 60 (Figure 1c).

The most substantial stabilization was observed for *L. delbrueckii* subsp. *bulgaricus* DSM 20081. While the control group showed a steep viability loss, dropping below 6.0 log cfu/mL just before day 55, the samples supplementation with 2.5% and 5.0% almond skin exhibited significantly lower death rates, maintaining viability above 6.5 log cfu/mL even at the end of the observation period (Figure 1d).

*S. thermophilus* DSM 20479 exhibited a marked decline in viability in the control group, with counts falling to around 6.0 log cfu/mL around day 70. However, supplementation with 2.5% almond skin significantly mitigated this decline, maintaining viability above 6.3 log cfu/mL up to around day 60. Even greater stabilization was achieved with higher almond skin concentrations (5.0% and 7.5%), with microbial counts consistently remaining near or above 6.7 log cfu/mL throughout the 90-day storage period (Figure 1e).

To quantitatively assess the protective effect of almond skin, two key parameters were calculated from the death kinetics: Sl-7 and Sl-6, defined as the time in days required to reach colony counts of 7 and 6 cfu/mL, respectively. These values, along with the death time (the predicted duration until microbial viability falls below a critical or detectable threshold), are represented in Table 5 for the five tested strains. The extended plateaus observed in Figure 1d,e for *L. delbrueckii* subsp. *bulgaricus* and *S. thermophilus* correspond directly to the high Sl-7, Sl-6 values and death times exceeding 500 days reported in Table 5, demonstrating the exceptional protective effect of almond skin supplementation at optimal concentrations (2.5–5.0%) for these strains. Conversely, the steeper decline curves observed in Figure 1a–c translate into shorter but significantly improved shelf-life parameters compared to controls.

No significant differences were observed at storage temperatures of 4 °C and 15 °C. In contrast, at 30 °C, the predicted Sl-7 and Sl-6 values were consistently higher in the almond skin-supplemented samples than in the controls.

*B. longum* DSM 20219 was the only strain for which the Sl-7, Sl-6, and death time values remained statistically similar across all treatments, showing no significant influence of almond skin supplementation.

For *L. acidophilus* DSM 20079, all almond skin concentrations significantly extended the predicted shelf life (Sl-7 and Sl-6) compared to the control. Sl-7 increased from 10.15 ± 4.02 days (CNT) to over 26 days across the supplemented groups. Sl-6 values more than doubled, ranging from 60.88 to 68.51 days, although death time remained statistically unchanged.

*L. plantarum* DSM 1055 showed a highly significant positive response to almond skin supplementation. The 2.5% level yielded the longest Sl-7 (35.67 ± 2.22 days) and death time (357.10 ± 38.51 days) values, representing a significant improvement over the control. Higher supplementation levels (5.0% and 7.5%) still outperformed the control but showed a decrease in Sl-6 and death time compared to 2.5%.

*L. delbrueckii* subsp. *bulgaricus* DSM 20081 demonstrated the most pronounced improvement, with 2.5% and 5.0% almond skin concentrations significantly extending both Sl-7 and Sl-6 times relative to the control group. Notably, the predicted death times for these two groups exceeded the observation period (>500 days), indicating superior protection. As with *L. plantarum*, higher concentrations (7.5%) resulted in a significant reduction in Sl-7, Sl-6, and death time compared to lower concentrations, suggesting a possible inhibitory effect at higher levels.

Finally, the viability of *S. thermophilus* DSM 20479 also increased significantly with supplementation. All treated groups showed considerably longer Sl-7and Sl-6 values. The predicted death time for all treatments, including the control group, was above 500 days.

Based on these results, a concentration of 3.5% almond skin was pragmatically selected for the subsequent biscuit formulation. While 2.5% showed the most pronounced protective effects for some strains (*L. plantarum* and *L. delbrueckii* subsp. *bulgaricus*), the selection of 3.5% represents a compromise between maximizing probiotic protection and ensuring sufficient polyphenolic content to potentially modulate gut microbiota, while avoiding the inhibitory effects observed at 7.5%. This intermediate concentration also provided optimal dough rheology and printability in preliminary formulation trials.

### 3.3. Effect of Almond Skin on Human Gut Microbiota Model

#### 3.3.1. Chemical and Bioactive Characterization of 3D-Printed Biscuits

Following the findings from the first phase, two 3D-printed biscuit formulations were developed: a control formulation containing only almond flour (F0), and another enriched with 3.5% almond skin powder (F1).

Biscuit containing almond skin exhibited a higher moisture content (4.480 ± 0.171 g/100 g) compared to the control without skin (3.612 ± 0.136 g/100 g). Similarly, water activity value was slightly elevated in the almond skin formulation (0.363 ± 0.002) relative to the control (0.359 ± 0.035) (Table 6). Macronutrient analysis indicated that the formulation without almond skin contained a marginally higher energy value (576.74 kcal/100 g) than that with almond skin (564.31 kcal/100 g). Protein contents were comparable in both formulations at 17.65 g and 17.24 g/100 g, respectively. A reduction in lipid content was observed in the almond skin biscuit (42.80 g/100 g) compared to the control (44.36 g/100 g), likely reflecting the compositional contribution of the skin. Total carbohydrate levels were similar between formulations (21.74 g/100 g for F1 vs. 21.93 g/100 g for F0). However, dietary fiber content was higher in the almond skin formulation (12.18 g/100 g) than in the control (10.19 g/100 g), consistent with the high fiber content of almond skin (62.6%, Table 3).

Regarding bioactive compounds, the enrichment with almond skin resulted in increased polyphenol content and antioxidant activity in both raw dough and baked biscuits (Table 6). In raw dough, F1 formulation showed TPC of 2.15 ± 0.04 mg GAE/g and DPPH activity of 2.07 ± 0.05 µmol TE/g, compared to F0 with TPC of 2.07 ± 0.01 mg GAE/g and DPPH of 1.62 ± 0.02 µmol TE/g. After baking, the enriched biscuits (F1) maintained higher bioactive compound levels, with TPC of 2.67 ± 0.04 mg GAE/g and DPPH of 1.92 ± 0.03 µmol TE/g, compared to the control biscuit (F0), which showed TPC of 2.38 ± 0.03 mg GAE/g and DPPH of 1.65 ± 0.01 µmol TE/g (Table 6).

#### 3.3.2. *In Vitro* Colonic Fermentation

**Figure 2:**
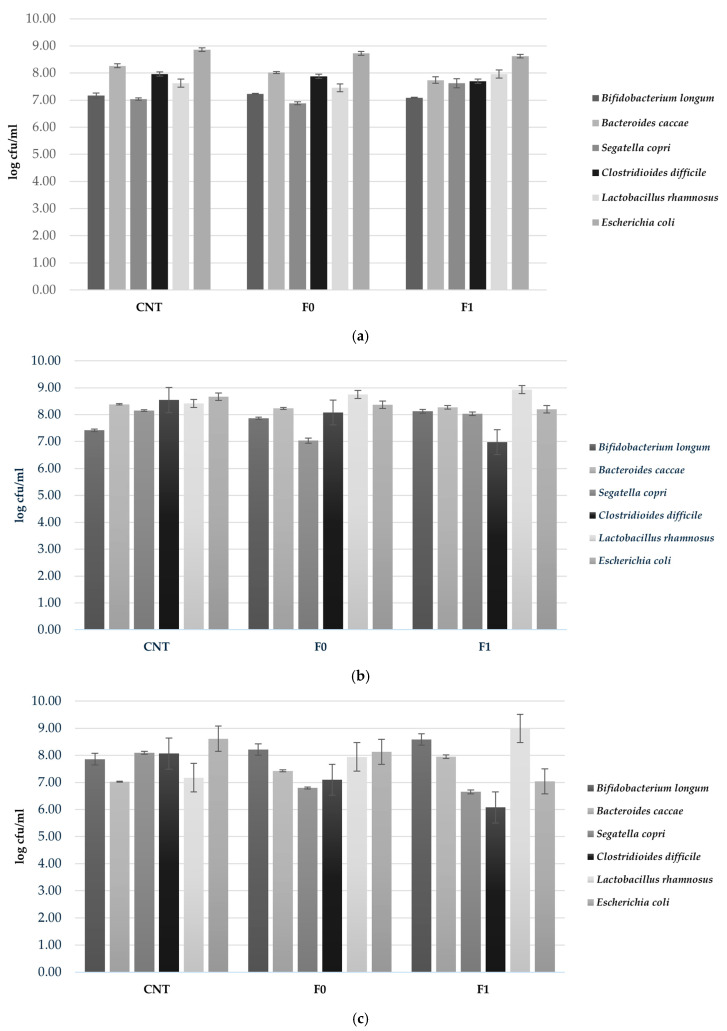
Viable bacterial counts (log cfu/mL) of probiotic strains during in vitro colonic fermentation of 3D-printed biscuits. (**a**) 6 h; (**b**) 24 h; (**c**) 48 h. Samples tested: CNT (water control); F0 (biscuits contain almond flour); F1 (biscuits enriched with 3.5% almond skin powder). Values represent mean ± standard deviation of three independent experiments.

**Figure 3:**
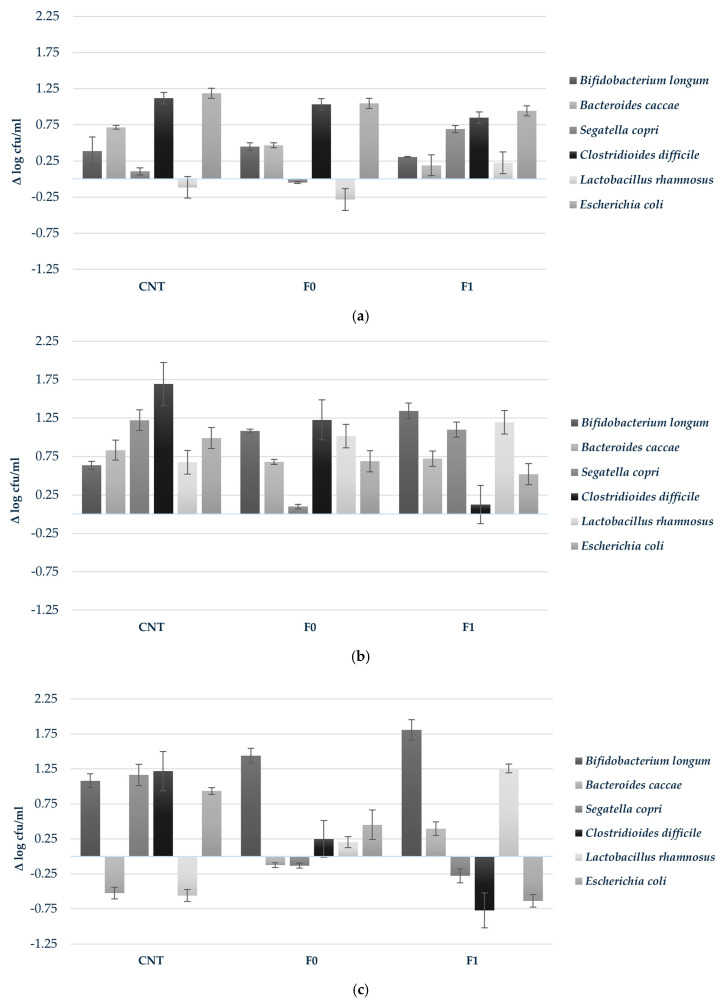
Growth response (Δ log cfu/mL) of probiotic strains during in vitro colonic fermentation of 3D-printed biscuits. (**a**) 6 h; (**b**) 24 h; (**c**) 48 h. Samples tested: CNT (water control); F0 (biscuits contain almond flour); F1 (biscuits enriched with 3.5% almond skin powder). Values represent mean ± standard deviation of three independent experiments.

The effects of two 3D-printed biscuit formulations (F0 and F1) on a human gut microbiota model (HGMM) were investigated using an in vitro colonic fermentation system, with sterile water as a negative control (CNT). Microbial populations were enumerated at 6, 24, and 48 h to assess temporal changes throughout fermentation (Figure 2). Absolute bacterial counts are presented in Figure 2 to enable assessment of probiotic viability thersholds (6–7 log CFU/mL), while delta values (Δ) showing growth trends relative to initial concentrations are presented in Figure 3 to emphasize prebiotic effects. At 6 h of fermentation, *B. longum* populations remained relatively stable across all treatments, with concentrations of 7.17, 7.23, and 7.08 log cfu/mL for CNT, F0, and F1, respectively. In contrast, *Bac. caccae* showed reduced abundance in the biscuit-supplemented samples, reaching 8.02 log cfu/mL in F0 and 7.74 log cfu/mL in F1 compared to 8.26 log cfu/mL in CNT. The beneficial species *L. rhamnosus* exhibited increased concentration in F1, attaining 7.96 log cfu/mL. Opportunistic pathogens *C. difficile* and *E. coli* displayed slightly lower concentrations in both F0 (7.88 and 8.72 log cfu/mL) and F1 (7.70 and 8.62 log cfu/mL) relative to CNT (7.97 and 8.86 log cfu/mL) (Figure 2a). As fermentation progressed to 24 h, pronounced shifts in microbial composition became evident (Figure 2b). *B. longum* experienced significant proliferation in F1, reaching 8.12 log cfu/mL and exceeding both F0 (7.86 log cfu/mL) and CNT (7.42 log cfu/mL). Similarly, *L. rhamnosus* achieved its maximum concentration in F1 (8.93 log cfu/mL). Conversely, potentially pathogenic species showed marked suppression in the almond skin-enriched formulation, with *C. difficile* declining to 6.98 log cfu/mL compared to 8.54 log cfu/mL in CNT. Both *E. coli* and *S. copri* exhibited comparable trends, decreasing to 8.20 and 8.03 log cfu/mL in F1, respectively, while maintaining higher levels in CNT (8.67 and 8.15 log cfu/mL) (Figure 2b). By the conclusion of the fermentation period (48 h), the prebiotic-like effects of F1 became increasingly pronounced. *B. longum* and *L. rhamnosus* sustained elevated concentrations of 8.59 and 8.99 log cfu/mL in F1, substantially exceeding those observed in both CNT (7.86 and 7.17 log cfu/mL) and F0 (8.22 and 7.94 log cfu/mL). Most remarkably, pathogenic bacteria were strongly inhibited in F1, with *C. difficile* and *E. coli* counts approximately two log units lower than CNT values (6.08 and 7.04 log cfu/mL versus 8.07 and 8.61 log cfu/mL, respectively) (Figure 2c).

To provide a more comprehensive understanding of microbiota dynamics, delta values (Δ) were calculated as the change in bacterial abundance relative to initial levels, allowing assessment of growth trends independent of baseline variation (Figure 3). At 6 h, C. *difficile* exhibited rapid early-stage proliferation across all conditions (Δ +1.12, +1.03, and +0.85 log cfu/mL for CNT, F0, and F1, respectively). Selective stimulation was observed for *S. copri* in F1 (Δ +0.69 log cfu/mL), while *L. rhamnosus* demonstrated modest but exclusive growth in this formulation (Δ +0.22 log cfu/mL) (Figure 3a). At 24 h, *B. longum* showed substantial expansion, particularly in F1 (Δ +1.34 log cfu/mL), accompanied by continued selective enhancement of *L. rhamnosus* (Δ +0.22 log cfu/mL), an effect uniquely associated with the almond skin-enriched formulation (Figure 3b). By 48 h, the prebiotic effect intensified, with *B. longum* achieving a growth increment of Δ +1.81 log cfu/mL in F1, while *L. rhamnosus* exhibited robust proliferation (Δ +1.26 log cfu/mL). Simultaneously, pathogenic species were progressively suppressed, with *C. difficile* and *E. coli* showing reduction of Δ −0.77 and −0.64 log cfu/mL, respectively, in F1 compared to their initial concentrations (Figure 3c).

## 4. Discussion

This study investigated the prebiotic potential of almond by-product, specifically almond skin, through a two-phase experimental approach bridging compositional characterization with functional validation. The first phase focused on characterizing the nutritional and bioactive properties of dehydrated almond skin and assessing its capacity to support probiotic viability under storage conditions. Building on this, the second phase evaluated the functional efficacy of 3D-printed biscuits enriched with almond skin through in vitro digestion and colonic fermentation using a model human gut microbiota. Our findings support the potential of almond skin for sustainable valorization as functional food ingredients that beneficially modulate gut microbiota and open new avenues for personalized nutrition development.

Compositional analysis confirmed that almond skin is a nutrient-dense by-product, particularly rich in dietary fiber (62.20 ± 12.10%), placing it among the highest plant-based fiber sources for food applications. This value aligns with and exceeds prior reports documenting total dietary fiber content ranging from 45% to 60% in various almond skin preparations [3,23]. The predominance of insoluble fibers such as cellulose, hemicellulose, and lignin may contribute to its prebiotic functionality by resisting digestion in the upper gastrointestinal tract and serving as a fermentable substrate for colonic microbiota [24,25]. These polysaccharides may favorably affect gut microbial communities, potentially promoting SCFA production, which plays a critical role in intestinal health and systemic metabolism [26]. However, SCFA production was not directly measured in this study. In addition to fiber, the fatty acid profile observed was dominated by unsaturated fatty acids (~86.90% of total fat), with oleic acid and linoleic acid as major components. This profile corresponds with recent studies highlighting the health benefits of almond lipids [3,7]. The high oleic acid content is consistent with the fatty acid composition reported for other almond-derived products [27,28]. The substantial polyphenol content (38.32 ± 0.04 mg GAE/g) confers antioxidant, antimicrobial, and prebiotic properties, corroborated by DPPH assays, indicating potential for oxidative stress mitigation [29,30,31]. The polyphenolic fraction of almond skin is known from literature to be rich in proanthocyanidins and flavonoids [3], which may contribute to the observed prebiotics effects. Moreover, significant zinc and iron levels were detected in almond skin.

The comparative analysis between almond flour and almond skin revealed a striking 15-fold difference in polyphenol content, definitively establishing almond skin as the primary reservoir of bioactive compounds in almonds. This finding is consistent with the well-documented phenomenon of bioactive compound concentration in plant seed coats and outer tissues, where polyphenols serve protective functions against environmental stressors, UV radiation, and pathogen attack [23]. The substantially lower TPC value in almond flour (2.57 mg GAE/g) compared to almond skin (38.32 mg GAE/g) confirms that removal of the skin during conventional almond processing results in significant loss of health-promoting compounds, reinforcing the value of skin recovery and valorization as functional food ingredients.

The viability study revealed strain-specific protective effects of almond skin supplementation during storage, with supplementation extending viability beyond the critical 6.0 log cfu/mL threshold for probiotic efficacy [32,33]. This protection likely stems from the synergistic action of fiber buffering pH changes, polyphenol antioxidant activity reducing oxidative damage, and fermentable carbohydrates sustaining probiotic metabolism [34,35,36]. Notably, *L. delbrueckii* subsp. *bulgaricus* and *L. plantarum* showed exceptional stabilization, with predicted death times exceeding 500 days at 2.5–5.0% almond skin, while a decrease at 7.5% may reflect several factors, including potential antimicrobial effects of high polyphenol concentrations, osmotic stress, or changes in matrix properties affecting nutrient availability [37,38]. The precise mechanisms underlying this dose-dependent response were not investigated in this study. These findings justified choosing 3.5% almond skin for biscuit formulation as a balanced prebiotic concentration that maximizes protective effects while avoiding inhibitory thresholds. Interestingly, the stable viability of *B. longum* across treatments likely reflects its inherent stress tolerance and metabolic specialization, consistent with its well-characterized oxidative stress defenses and substrate utilization capabilities [39].

Building upon the established prebiotic properties, the use of 3D food printing to produce almond skin-enriched biscuits represents a strategic innovation enabling personalized functional foods through precise control of nutrient and bioactive compound distribution [40,41]. This technological approach allows for customization of fiber and polyphenol doses according to individual nutritional requirements, representing a significant advancement over traditional food processing methods.

Compared to controls, enriched biscuits showed increased moisture and water activity due to fiber’s hygroscopic nature, along with a compositional shift favoring carbohydrates and reduced lipids [28]. The analysis of bioactive compounds throughout the manufacturing process provided critical insights into the stability and retention of polyphenols during thermal treatment. The comparative evaluation of raw dough and baked biscuits revealed that thermal processing at 150 °C for 18 min resulted in a concentration effect rather than degradation, with TPC values increasing from dough to biscuit in both formulations. This increase can be attributed to moisture loss during baking, leading to a concentration of phenolic compounds in the final product. Importantly, the enriched formulation (F1) maintained consistently higher TPC and DPPH values than the control (F0) both before and after baking, demonstrating that the bioactive advantage conferred by almond skin addition is preserved throughout processing. The retention of polyphenol content and antioxidant activity post-processing indicates the protective role of the biscuit matrix against thermal degradation, congruent with literature on plant by-product enriched bakery products [24,42,43]. This stability is particularly relevant for functional food applications, as it ensures that bioactive compounds remain available for potential gut microbiota fermentation and subsequent health benefits. The preserved antioxidant capacity in the final product further supports the hypothesis that observed prebiotic effects in the colonic fermentation model can be attributed not only to the fiber fraction but also to the synergistic action of phenolic compounds present in almond skin.

To address the experimental objectives with controlled complexity, a minimal human microbiota model composed of 6 representative strains was employed. Minimal consortia consisting of 6 to 12 species are commonly used in mechanistic studies to allow precise evaluation of microbial interactions and substrate utilization, while larger models with over 20 species better mimic the resilience and functional diversity of the in vivo gut microbiota [44,45,46]. The six strains comprising the HGMM were selected based on three criteria: (1) representation of the major microbial phyla found in the human gut (Firmicutes, Bacteroidetes, Actinobacteria, and Proteobacteria), (2) documented roles in gut health (*B. longum*, *L. rhamnosus* as beneficial commensals) and disease (*C. difficile*, *E. coli* as opportunistic pathogens), and (3) established use in previous minimal consortium studies investigating prebiotic effects [15,16,17]. This approach captures essential taxonomic and functional characteristics relevant to gut ecology while enabling controlled mechanistic investigation. However, this simplified model, while enabling mechanistic investigation of specific microbe substrate interactions, cannot fully represent the complexity, diversity, and functional redundancy of the human gut microbiota. This approach allows for controlled investigation of prebiotic effects while acknowledging that in vivo responses may differ due to the presence of additional microbial species and host–microbe interactions. In vitro colonic fermentation demonstrated selective stimulation of key beneficial genera, notably *B. longum* and *L. rhamnosus*, with significant suppression of pathogenic strains including *C. difficile* and *E. coli*. The bifidogenic effect likely results from the enzymatic machinery of *Bifidobacterium* to hydrolyze complex carbohydrates and metabolize polyphenols, processes that typically lead to increased SCFA production and may enhance intestinal barrier integrity [47]. However, SCFA production was not quantified in this study and remains to be confirmed in future work. *Lactobacillus* benefits also stem from competitive exclusion, production of antimicrobials such as bacteriocins, and immunomodulatory effects [48]. Simultaneously, pathogen suppression may be attributable to acidification (likely from SCFA production) of the colonic environment and direct antimicrobial effects of almond skin polyphenols disrupting microbial membranes and inhibiting virulence factors [38,49,50]. This dual mechanism, prebiotic stimulation of beneficial bacteria combined with antimicrobial suppression of pathogens, represents a powerful approach for gut health modulation [47]. The observed prebiotic effects are likely attributable to the combined action of dietary fibers and polyphenolic compounds present in almond skin, though the precise contribution of each component following gastrointestinal digestion was not quantified in this study. The selective growth of *S. copri* observed in the almond skin-enriched formulation warrants careful interpretation given the documented strain-level functional variability of this species, with different isolates showing distinct polysaccharide utilization profiles and potentially opposing health effects [51,52]. More importantly, the consistent and robust beneficial effects observed for *B. longum* and *L. rhamonosus*, coupled with the marked suppression of the opportunistic pathogens *C.difficile* and *E. coli*, strongly support the prebiotic potential of almond skin in promoting a beneficial gut microbiota composition.

The selective modulation of gut microbiota by almond skin-enriched foods presents promising avenues for managing dysbiosis-related conditions such as metabolic syndrome, inflammatory bowel disease, and obesity, though clinical validation remains essential [53,54]. The integration of sustainable by-product valorization with personalized nutrition through 3D printing technology represents a novel approach that addresses both environmental sustainability and individual health optimization. While the HGMM consortium employed in this study provides valuable mechanistic insights into prebiotic effects of almond skin, several limitations must be acknowledged. The use of six bacterial strains, although representative of major gut phyla and pathogenic species, cannot capture the full complexity, metabolic diversity, and functional redundancy of the human gut microbiota, which comprises hundreds of species with intricate ecological interactions. The selective effects observed in this simplified model may be amplified, attenuated, or modified in mor complex microbial communities where competitive and synergistic interactions are more pronounced. Furthermore, the in vitro fermentation system, while following the standardized INFOGEST protocol, lacks host factors such as mucus production, immune responses, and gut transit dynamics that significantly influence microbiota composition and activity in vivo. Therefore, while our findings demonstrate the potential of almond skin enriched biscuits to beneficially modulate gut microbiota, validation through more complex in vitro models and ultimately through human intervention trails is essential to confirm these effects and establish clinical relevance. Additionally, we did not quantify polyphenolic compounds or SCFA production in the fermentation system. Therefore, while the prebiotic effects observed are likely associated with the bioactive compounds present in almond skin, we cannot establish direct quantitative relationships between specific compound concentrations and microbiota modulation.

## 5. Conclusions

This study confirms that dehydrated almond skin is a nutrient-rich by-product characterized by high dietary fiber content and a favorable fatty acid profile dominated by unsaturated fats, mainly oleic and linoleic acids. The bioactive compound analysis revealed significant polyphenol concentrations and antioxidant capacity, supporting its potential as a functional food ingredient. Almond skin supplementation significantly enhanced the viability of various probiotic strains during storage, with an optimal concentration around 2.5–3.5% that balances microbial protection without inhibitory effects. Incorporation of this ingredient into 3D-printed biscuit formulations preserved bioactive properties and modified macronutrient profiles, favoring fiber and carbohydrate content. In vitro fermentation using a minimal human gut microbiota model showed that almond skin-enriched biscuits selectively stimulated beneficial bacteria such as *B. longum* and *L. rhamnosus* while suppressing pathogenic species including *C. difficile* and *E. coli*. This dual prebiotic and antimicrobial effect underscores the ingredient’s promise for gut microbiota modulation. While this work demonstrates innovative use of almond skins in 3D food printing to create functional foods with personalized nutrition potential, this study is limited by a simplified in vitro model. Future in vivo and clinical studies are necessary. Overall, almond skin is a sustainable ingredient that aligns with the principle of the circular economy while advancing health-oriented food innovation.

## Figures and Tables

**Table 1:** List of the bacterial strains selected from the literature to construct the human gut microbiota model (HGMM).

Phylum	Strains	Medium
*Actinobacteria*	*Bifidobacterium longum* DSM 20219	MRS Agar + 0.5% L-cystein
*Bacteroidetes*	*Bacteroides caccae* DSM 19024	Columbia blood medium Agar
	*Segatella copri* DSM 18205	Schaedler Anaerobe Agar
*Firmicutes*	*Clostridiodies difficile* DSM 27543	Anaerobe Basal Agar
	*Lactobacillus rhamnosus* DSM 20021	MRS Agar
*Proteobacteria*	*Escherichia coli* DSM 1058	Violet Red Bile Glucose Agar (VRBGA)

**Table 2:** Simulated digestion conditions according to INFOGEST protocol.

Simulated Fluid	Electrolyte	Enzymes	Duration
Simulated salivary fluid (SSF)	KCl, KH_2_PO_4_, NaHCO_3_, MgCl_2_(H_2_O)_6_, (NH_4_)_2_CO_3_, HCl, CaCl_2_(H_2_O)_2_	Salivary amylase (75 U/mL)	2 min
Simulated gastric fluid (SGF)	KCl, KH_2_PO_4_, NaHCO_3_, MgCl_2_(H_2_O)_6_, NaCl, (NH_4_)_2_CO_3_, HCl, CaCl_2_(H_2_O)_2_	Pepsin (2000 U/mL)	120 min
Simulated intestinal fluid (SIF)	KCl, KH_2_PO_4_, NaHCO_3_, MgCl_2_(H_2_O)_6_, NaCl, HCl, CaCl_2_(H_2_O)_2_	Bile salt (10 mM), pancreatin (100 U/mL)	120 min

**Table 3:** Nutritional composition of dehydrated almond skin. TPC, total phenol content; DPPH, 2,2-diphenyl-1-picrylhydrazyl. Values represent the standard deviation of three lots of almond skin.

Parameters	Dehydrated Almond Skin
Total Dietary Fiber (%)	62.60 ± 12.10
Total Fat (%)	16.10 ± 0.60
Protein (%)	11.75 ± 1.40
Manganese (mg/kg)	53.19 ± 9.36
Selenium (mg/kg)	0.05 ± 0.02
Zinc (mg/kg)	83.70 ± 13.80
Iron (mg/kg)	62.00 ± 11
Copper (mg/kg)	8.19 ± 1.91
TPC (mg GAE/g)	38.32 ± 0.04
DPPH (µmol TE/g)	5.08 ± 0.02

**Table 4:** Fatty acid composition of dehydrated almond skin expressed as a relative percentage. Values represent the standard deviation of three lots of almond skin.

Fatty Acid Composition (%)	
Butyric acid (C4:0)	<0.05
Caproic acid (C6:0)	<0.05
Caprylic acid (C8:0)	<0.05
Capric acid (C10:0)	<0.05
Undecanoic acid (C11:0)	<0.05
Lauric acid (C12:0)	<0.05
Tridecanoic acid (C13:0)	<0.05
Myristic acid (C14:0)	<0.05 ± 0.01
Myristoleic acid (C14:1)	<0.05
Pentadecanoic acid (C15:0)	0.11 ± 0.02
Palmitic acid (C16:0)	10.05 ± 1.42
Palmitoleic acid (16:1)	0.69 ± 0.11
Heptadecanoic acid (C17:0)	0.14 ± 0.03
Heptadecenoic acid (C17:1)	0.07 ± 0.03
Stearic acid (C18:0)	2.55 ± 0.35
Elaidic acid (C18:1)	<0.05
Oleic acid (C18:1)	47.14 ± 6.36
Trans-9-trans-12 Octadecadienoic acid (Trans-linoleic acid) (C18:2)	<0.05
Linoleic acid (omega-6) (C18:2)	38.34 ± 5.18
Gamma-linolenic acid (omega-6) (C18:3)	<0.05
Alpha-linolenic acid (omega-3) (C18:3)	0.64 ± 0.11
Arachidic acid (C20:0)	0.20 ± 0.04
Eicosenoic acid (C20:1)	<0.05
Eicosadienoic acid (omega-6) (C20:3)	<0.05
Heneicosanoic acid (C21:0)	<0.05
Cis-8-eicosatrienoic acid (omega-6) (C20:3)	<0.05
Arachidonic acid (omega-6) (C20:4)	<0.05
Cis-11-eicosatrienoic acid (omega-3) (C20:3)	<0.05
Behenic acid (C22:0)	<0.05
Eicosapentaenoic acid (omega-3) (C20:5)	<0.05
Erucic acid (C22:1)	<0.05
Docosadienoic acid (C22:2)	<0.05
Tricosanoic acid (C23:0)	<0.05
Lignoceric acid (C24:0)	<0.05
Nervonic acid (C24:1)	<0.05
Docosahexaenoic acid (omega-3) (C22:6)	<0.05
Sum of saturated fatty acids	13.10 ± 1.46
Sum of monounsaturated fatty acids	47.92 ± 6.36
Sum of polyunsaturated fatty acids	38.98 ± 5.18

**Table 5:** Predicted shelf life and death time of *Lactobacillus acidophilus*, *Lactobacillus delbruekii* subsp. *bulgaricus*, *Streptococcus thermophilus*, *Bifidobacterium longum*, and *Lactiplantibacillus plantarum*, inoculated in Reconstituted Skim Milk, added with almond skin (2.5–5.0–7.5%) and stored at 30 °C for 90 days. Values represent the standard deviation of three lots of almond skin. Sl-7, time (day) to attain a cell count of 7 log CFU/mL; Sl-6, time (day) to attain a cell count of 6 log cfu/mL; d.t., death time (day) of the microbial population. Letters indicate significant differences for each strain (one way ANOVA and Tukey’s test).

		Sl-7	Sl-6	d.t
Strain	Sample	(Day)	(Day)	(Day)
*Bifidobacterium longum* DSM 20219	CNT	29.16 ± 4.30 ^a^	54.01 ± 3.69 ^a^	283.21 ± 21.37 ^a^
	2.5%	30.78 ± 8.84 ^a^	58.03 ± 3.96 ^a^	319.17 ± 19.69 ^a^
	5.0%	38.86 ± 2.48 ^a^	67.68 ± 4.30 ^a^	305.23 ± 27.17 ^a^
	7.5%	31.27 ± 4.71 ^a^	58.42 ± 8.40 ^a^	314.77 ± 34.68 ^a^
*Lactobacillus acidophilus* DSM 20079	CNT	10.15 ± 4.02 ^a^	30.38 ± 7.43 ^a^	380.74 ± 25.62 ^a^
	2.5%	30.60 ± 9.61 ^b^	68.51 ± 4.46 ^b^	438.25 ± 25.35 ^a^
	5.0%	26.20 ± 4.75 ^b^	60.88 ± 6.63 ^b^	424.17 ± 27.94 ^a^
	7.5%	27.97 ± 1.17 ^b^	61.20 ± 7.48 ^b^	371.32 ± 20.87 ^a^
*Lactiplantibacillus plantarum* DSM 1055	CNT	17.74 ± 5.19 ^a^	33.43 ± 4.49 ^a^	193.08 ± 15.82 ^a^
	2.5%	35.67 ± 2.22 ^b^	67.86 ± 2.30 ^b^	357.10 ± 38.51 ^b^
	5.0%	27.80 ± 7.39 ^b^	52.40 ± 3.80 ^c^	232.40 ± 24.71 ^c^
	7.5%	25.02 ± 6.49 ^b^	47.16 ± 3.42 ^c^	243.06 ± 14.39 ^c^
*Lactobacillus delbruekii* subsp. *bulgaricus* DSM 20081	CNT	32.80 ± 6.43 ^a^	57.50 ± 5.07 ^a^	235.21 ± 8.09 ^a^
	2.5%	92.89 ± 7.95 ^b^	167.47 ± 4.61 ^b^	>500
	5.0%	81.49 ± 2.46 ^b^	141.91 ± 1.36 ^c^	>500
	7.5%	48.80 ±1.86 ^c^	86.13 ± 4.08 ^d^	357.05 ± 20.53 ^b^
*Streptococcus thermophilus* DSM 20479	CNT	11.97 ± 3.55 ^a^	68.79 ± 3.87 ^a^	>500
	2.5%	25.29 ± 2.82 ^b^	145.31± 9.62 ^b^	>500
	5.0%	16.08 ± 7.49 ^b^	92.39 ± 7.20 ^c^	>500
	7.5%	17.01 ± 4.08 ^b^	97.74 ± 8.45 ^c^	>500

**Table 6:** Nutritional and physical characteristics of raw dough and 3D-printed biscuits. Samples tested: F0 (biscuits contain almond flour); F1 (biscuits enriched with 3.5% almond skin powder). TPC, total phenol content; DPPH, 2,2-diphenyl-1-picrylhydrazyl. Values represent mean ± standard deviation.

Parameter	F0	F1	F0 Dough	F1 Dough
Energy (kcal/100 g)	576.74 ± 1.30	564.31 ± 1.18	-	
Proteins (g/100 g)	17.65 ± 1.40	17.24 ± 1.35	-	
Lipids (g/100 g)	44.36 ± 0.70	42.80 ± 0.65	-	
Carbohydrates (g/100 g)	21.93 ± 0.80	21.74 ± 0.70	-	
of which sugars (g/100 g)	20.66 ± 0.22	20.51 ± 0.30	-	
Dietary fiber (g/100 g)	10.19 ± 0.50	12.18 ± 0.40	-	
TPC (GAE/g)	2.38 ± 0.03	2.67 ± 0.04	2.07 ± 0.01	2.15 ± 0.04
DPPH (µmol TE/g)	1.65 ± 0.01	1.92 ± 0.03	1.62 ± 0.02	2.07 ± 0.05
Water activity (aw)	0.359 ± 0.03	0.363 ± 0.02	-	-
Moisture content (g/100 g)	3.61 ± 0.14	4.48 ± 0.17	-	-

## Data Availability

The original contributions presented in the study are included in the article. Further inquiries can be directed to the corresponding author.
